# Association between the metabolic score for insulin resistance (METS-IR) and estimated glomerular filtration rate (eGFR) among health check-up population in Japan: A retrospective cross-sectional study

**DOI:** 10.3389/fendo.2022.1027262

**Published:** 2022-12-16

**Authors:** Gailing Liu, Lin Tao, Qing Zhu, Xiaojing Jiao, Lei Yan, Fengmin Shao

**Affiliations:** Department of Nephrology, People’s Hospital of Zhengzhou University, He’nan Provincial People’s Hospital, He’nan Provincial Key Laboratory of Kidney Disease and Immunology, Zhengzhou, China

**Keywords:** metabolic score for insulin resistance (METS-IR), estimated glomerular filtration rate (eGFR), insulin resistance, insulin sensitivity, chronic kidney disease (CKD)

## Abstract

**Aim:**

This study aimed to investigate the relationship between a new metric—metabolic score for insulin resistance (METS-IR)—and estimated glomerular filtration rate (eGFR) among Japanese participants who underwent health check-ups.

**Methods:**

We conducted a cross-sectional study that involved participants in a medical health screening program, which was conducted at the Medical Health Check-up Center in Japan. This retrospective study examined the relationship between METS-IR and eGFR among 881 individuals that joined the program between March 1, 2004, and December 31, 2012. Covariates consisted of serum laboratory tests and lifestyle questionnaires. Multivariate linear regression analysis was used to explore the association between METS-IR and eGFR. In addition, subgroup and interaction analyses were done based on age, sex, body mass index (BMI), alcohol use, smoking status, and hyperuricemia.

**Results:**

A total of 881 individuals participated in this study. High METS-IR was highly linked with reduced eGFR (adjusted β = -5.04, 95% confidence interval (CI): -7.65 to -2.43), while METS-IR was utilized as a categorical variable inside the multiple regression analysis. A decrease in eGFR of 2.54 units was reported for every 10-unit rise in METS-IR (adjusted β = -2.54, 95% CI: -4.04 to -1.05, *P*-value = 0.001). Stratified analysis suggested no marked interaction between METS-IR and eGFR across age, sex, BMI, and alcohol consumption groups. However, there was an indication of interaction between METS-IR level, smoking status (*P*-value = 0.001), and uric level (*P*-value = 0.011) on eGFR decrease.

**Conclusions:**

METS-IR is remarkably associated with eGFR among the participants who underwent health check-ups in Gifu, Japan. Although more studies are required to prove it, METS-IR could be applied as a monitoring index for early screening, primary prevention, and diagnostic and treatment management strategies for chronic kidney disease.

## 1 Introduction

Chronic kidney disease (CKD) is a threat to human health and a social and economic burden that affects people worldwide (1). CKD is symptomatic until the disease stage advances. Furthermore, CKD has been linked to an increased risk of cardiovascular illness (2). As a result, identifying and treating CKD in its early stages are critical for avoiding end-stage renal failure, cardiovascular disease, and mortality. In 2019, Kidney Disease: Improving Global Outcomes (KDIGO) proposed that screening, risk stratification, and therapy for CKD in at-risk individuals should be initiated immediately, preferably in primary health facilities or the community (3). Screening for CKD and catching it early can greatly decrease the risk of death and illness from CKD and its comorbidities, such as heart disease (4, 5). In addition, KDIGO recommended using the estimated glomerular filtration rate (eGFR) as part of the first line of screening for CKD (3).

Insulin resistance (IR) is a state of diminished response to insulin in target cells and an important pathogenic mechanism for the development of metabolic diseases. It is present in many chronic diseases, including diabetes, cardiovascular disease, hypertension, and CKD (6, 7). Furthermore, several investigations revealed that being overweight and having metabolic syndrome (MS) was significantly linked to a moderate decline in eGFR (7–12). Therefore, we hypothesized that there might be an association between IR or related surrogate markers and eGFR.

The metabolic insulin resistance score (METS-IR) is a new metric for evaluating IR and assessing cardiometabolic risk in healthy and high-risk populations (13). As the gold standard for assessing insulin resistance (IR), hyperinsulinemic euglycemic clamp (HEC) is a steady-state technique but is limited in large-scale clinical studies and epidemiological investigations because of its invasive, laborious, expensive, and complex nature (14, 15). Furthermore, insulin-based IR metrics, such as homeostatic model assessment (HOMA), also have limitations in terms of accuracy and stability (13, 15). Recently, some IR alternatives that are not based on insulin assays, such as triglyceride-glucose (TyG) index (16) and METS-IR (13, 17), have become increasingly attractive. In the study by Bello-Chavolla OY et al. (13), METS-IR was validated in three samples: one with education, health, and care plan (HEC) data, one with modified frequently sampled intravenous glucose tolerance test (FSIVGTT) data, and one with a large cohort against HOMA-IR. Compared with that of HEC and the SI index generated from the FSIVGTT, METS-IR showed a stronger association with the M-value adjusted by fat-free mass and diagnostic performance to identify reduced insulin sensitivity (IS). In addition, METS-IR was effective in identifying people with early hypertension (17, 18) and was more valid in predicting the risk of type 2 diabetes (13), ischemic heart disease (19) and coronary heart disease (20). These studies primarily concentrated on the association of IR with hypertension or cardiovascular disease. However, few studies have addressed the correlation between METS-IR and eGFR. Therefore, this cross-sectional study aimed to explore whether there was a clear association between METS-IR and eGFR in the Japanese medical examination population.

## 2 Methods

### 2.1 Study design and population

We designed a cross-sectional study that involved participants in a medical health screening program performed at the Medical Health Check-up Center in Japan. The program aims to enhance community health by detecting and evaluating risk factors for chronic diseases at an early stage. This medical screening service, named “human dock,” is very well received in Japan.

The data were extracted from the DRYAD database (http://www.Datadryad.org/). All researchers can get the Dryad data package containing the information for free (Fukuda, Takuya, et al., 2016) (21). Information about the work by Fukuda, Takuya, et al. was gathered using thorough citations (Dryad, https://doi.org/10.5061/dryad.m484p).

People who had their brachial-ankle pulse wave velocity checked as part of a health screening program at Murakami Memorial Hospital from March 2004 to December 2012 were asked to participate in the study. The Murakami Memorial Hospital’s ethics committee approved the study, and all participants provided informed consent before participating in the study (22). This study was conducted according to the “Guidelines for Strengthening Reports of Observational Studies in Epidemiology (STROBE)” (23).

The study excluded pregnant women, people taking oral contraceptives and hormones, those who were detected as positive for hepatitis B antigen and/or hepatitis C antibodies, and those with an ankle-brachial index below 0.95 (21). In the original article, a total of 912 participants participated in the study according to the nadir criteria (21). We further directly deleted 30 missing values for alcohol consumption, exercise, and fatty liver, as well as 1 with abnormal triglyceride (TG), resulting in 881 participants included in our study.

### 2.2 Data collection and measurements

The health check-up included height, weight, blood pressure, blood biochemical index measurement, urine analysis, routine blood test, blood glucose, blood lipids, and ultrasound examination (22). The data were from the publicly available Dryad database. The detailed data collection methods were outlined in the original work by Takuya Fukuda et al. (21). Ultrasound examinations were performed by skilled specialists using the Aloka SSD-650CL (Aloka Co, Ltd, Tokyo, Japan) to diagnose the presence or absence of fatty liver based on examination prompts. A judgment against fatty liver was also made by another physician on the ultrasound photographs without consulting the participants’ personal information (24). Fatty liver was defined according to the two necessary conditions of liver echo contrast and brightness (25). The body mass index (BMI) was determined by dividing the participant’s height in meters squared by their body weight in kg. The formula for METS-IR was Ln [(2 × fasting glucose (mg/dL)) + fasting TG (mg/dL)] × BMI (kg/m^2^))/(Ln [HDL-c (high‐density lipoprotein cholesterol) (mg/dL)]). eGFR was computed using the Japanese Society of Nephrology formula: eGFR = 194 × Cr−1.094 × age−0.287 (mL/min/1.73 m^2^) for males, while eGFR was multiplied by a correction factor of 0.739 for females. Serum uric acid levels > 420 mmol/L in men or 360 mmol/L in women were considered as having hyperuricemia (HUA) (26).

The same skilled team of interviewers distributed a standardized questionnaire to each participant. By asking the participants how many and what kind of alcoholic beverages they had each week during the previous month, researchers were able to estimate the average amount of alcohol they drank each week. Total weekly alcohol consumption was counted in grams and then classified as follows: nil or little (40 g/week); light (40–140 g/week); moderate (140–280 g/week); excessive (> 280 g/week) (24, 27, 28). Additionally, two categories were defined by their smoking habits: none or past and current smokers (24). Participants listed the kind, length, and frequency of the sports or leisure activities they engaged in on the survey (29). We classified participants as regular exercisers if they engaged in any type of sport on a regular basis, at least once per week (30).

### 2.3 Statistical analysis

Data were divided into categories and continuous variables. Student’s t-test compared continuous, normally distributed variables between groups. Non-normally distributed data were compared using the Wilcoxon rank-sum test and reported as the median interquartile range. Comparing categorical variables as percentages with the chi-square test. Kruskal–Wallis test or one-way analysis of variance analyzed differences between METS-IR index quartile groups. Multivariate regression was utilized to evaluate the relationship between METS-IR and eGFR. We calculated regression coefficients and a 95% confidence interval (CI). Due to the low number of missing values (3.2%), this study used direct deletion.

To investigate confounding, we added or removed covariates for correction in the linear regression models one by one and compared the corresponding effect values. Covariates with effect values changing by more than 10% were included for multi-model adjustment in our study (31), and aspartate aminotransferase (AST) and gamma-glutamyl transferase (GGT) were excluded. During covariate selection, variance inflation factor ≥ 5 indicated the presence of multicollinearity, and BMI, total cholesterol (TC), TG, high-density lipoprotein-cholesterol (HDL-c), and low-density lipoprotein-cholesterol (LDL-c) were excluded. Considering the previous literature reporting a possible association between IR and HUA (32, 33), we did not include uric acid as a variable for model adjustment. However, we performed a subgroup analysis by grouping uric acid levels to explore whether there was a link between METS-IR and eGFR in people with different uric acid levels. Age, sex, systemic blood pressure (SBP), diastolic blood pressure (DBP), ALT, fasting blood glucose (FPG), exercise, fatty liver, alcohol consumption, and smoking status were eventually included in the multi-model adjustment. We developed three models. Age and sex were taken into account in Model 1. Age, sex, SBP, DBP, ALT, and FPG levels were accounted for in Model 2. Model 3 was based on model 2 and lifestyle factors (exercise, fatty liver, alcohol consumption, and smoking status). Interaction and stratification analyses were performed based on age (< 65 or ≥ 65 years), sex (male or female), BMI (< 25 or ≥ 25 kg/m^2^), alcohol consumption, smoking status, and HUA (hyperuricemia or non-hyperuricemia). Stratified linear regression models were used for subgroup analysis. In addition, we used smoothed curve fitting (penalized spline method) to observe whether METS-IR had a nonlinear relationship with eGFR. For sensitivity analysis, METS-IR was also turned into a categorical variable, and the P trend was computed. R version 4.0.2 (http://www.R-project.org) and “Free Statistics software” were used for all statistical tests (version 1.6). P < 0.05 was regarded as being statistically significant.

## 3 Results

### 3.1 Baseline characteristics of the participants

**Figure 1:**
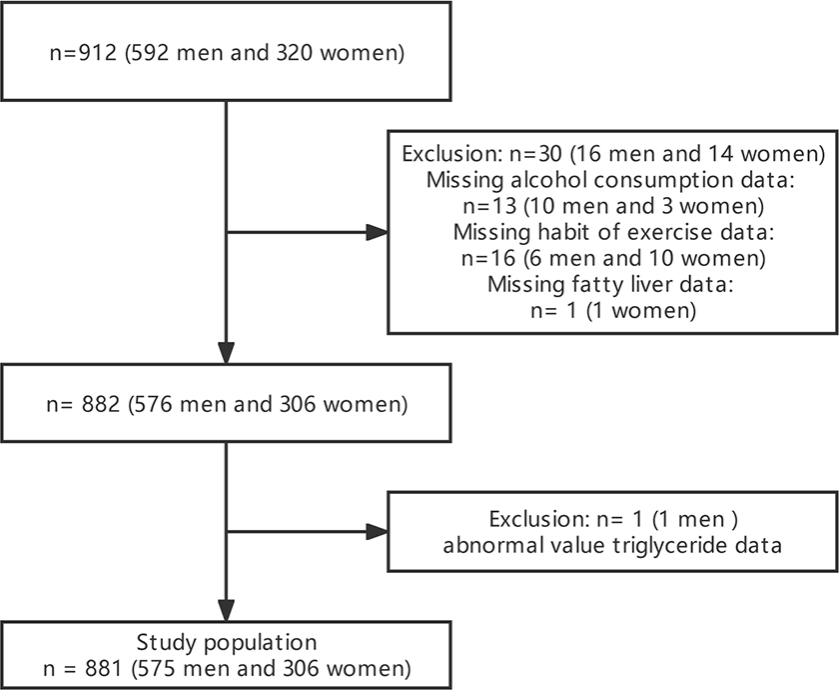
Flowchart of participant selection.

Between March 2004 and December 2012, 1,445 participants in the Murakami Memorial Hospital health check-up program had their medical records with clinical features examined. This study comprised 912 participants (592 men and 320 women). The detailed process of inclusion and exclusion was as described in the previous work of Fukuda et al. (21). After the direct deletion of 30 missing values for alcohol consumption, exercise, and fatty liver, as well as 1 abnormal TG, 881 participants were finally used for analyses. The flowchart for participant selection is presented in **Figure 1**.

Based on the quartile groups of the METS-IR, the baseline characteristics of the study participants are shown in **Table 1**. The average participant age was 51.1± 9.4 years, and 575 (65.3%) were men. Significant statistical differences were detected in sex, age, BMI, SBP, DBP, AST, ALT, GGT, FPG, UA, TG, HDL-c, LDL-c, alcohol consumption, smoking status, a habit of exercise, and fatty liver among different METS-IR index groups (all P-values < 0.05). Interestingly, male participants with high alcohol consumption, smoking, and fatty liver had significantly higher METS-IR. The opposite patterns were observed in the habit of exercise. Regular exercisers tended to have much lower METS-IR. This was consistent with our common knowledge as well as previous studies (17).

**Table 1:** Baseline characteristic of the study population according to METS-IR.

Variables	Total (n = 881)	Q1 (n = 222)	Q2 (n = 220)	Q3 (n = 220)	Q4 (n = 219)	*P* value
Sex, n (%)						< 0.001
Male	575 (65.3)	82 (36.9)	126 (57.3)	178 (80.9)	189 (86.3)	
Female	306 (34.7)	140 (63.1)	94 (42.7)	42 (19.1)	30 (13.7)	
Age, (years)	51.1 ± 9.4	50.8 ± 9.8	52.4 ± 9.1	51.2 ± 9.4	50.0 ± 9.3	0.049
BMI, (kg/m2)	23.1 ± 3.1	19.8 ± 1.4	22.3 ± 1.3	23.8 ± 1.5	26.7 ± 2.9	< 0.001
SBP, (mmHg)	120.2 ± 15.0	112.3 ± 13.1	118.2 ± 14.7	123.3 ± 14.4	127.3 ± 13.2	< 0.001
DBP, (mmHg)	76.1 ± 10.0	70.3 ± 8.9	74.6 ± 9.7	78.4 ± 9.4	81.3 ± 8.3	< 0.001
AST, (IU/L)	20.8 ± 8.1	19.4 ± 6.1	19.0 ± 5.8	20.9 ± 6.9	24.0 ± 11.5	< 0.001
ALT, (IU/L)	19.0 (14.0, 26.0)	15.5 (12.0, 19.0)	16.0 (13.0, 21.0)	20.0 (16.0, 27.0)	26.0 (20.0, 40.0)	< 0.001
GGT, (IU/L)	19.0 (14.0, 28.0)	14.0 (11.0, 18.0)	16.0 (13.0, 24.0)	21.0 (15.8, 33.0)	25.0 (19.0, 38.0)	< 0.001
FPG, (mg/dl)	98.1 ± 14.2	92.6 ± 8.1	95.7 ± 9.8	99.2 ± 18.5	105.0 ± 15.0	< 0.001
UA, (mg/dl)	5.2 (4.2, 6.1)	4.4 (3.6, 5.2)	5.0 (4.0, 5.8)	5.7 (4.7, 6.2)	6.0 (5.3, 6.9)	< 0.001
TC, (mg/dl)	209.5 ± 35.8	208.0 ± 37.7	206.4 ± 32.8	208.9 ± 35.4	214.9 ± 36.7	0.07
TG, (mg/dl)	80.0 (53.0, 123.0)	50.0 (37.0, 69.0)	65.0 (51.0, 88.0)	92.0 (70.0, 127.5)	141.0 (100.0, 192.5)	< 0.001
HDL-c, (mg/dl)	53.6 ± 14.6	66.8 ± 13.6	57.4 ± 11.4	48.4 ± 9.7	41.8 ± 9.2	< 0.001
LDL-c, (mg/dl)	127.9 ± 31.8	118.5 ± 32.9	125.6 ± 28.4	132.0 ± 31.2	135.5 ± 31.9	< 0.001
Alcohol consumption, n (%)						0.006
None or minimal	567 (64.4)	165 (74.3)	139 (63.2)	126 (57.3)	137 (62.6)	
Light	147 (16.7)	31 (14)	38 (17.3)	45 (20.5)	33 (15.1)	
Moderate	88 (10.0)	10 (4.5)	28 (12.7)	28 (12.7)	22 (10)	
Heavy	79 (9.0)	16 (7.2)	15 (6.8)	21 (9.5)	27 (12.3)	
Smoking status, n (%)						0.001
None or Past	690 (78.3)	187 (84.2)	183 (83.2)	164 (74.5)	156 (71.2)	
Current	191 (21.7)	35 (15.8)	37 (16.8)	56 (25.5)	63 (28.8)	
Habit of exercise, n (%)						0.002
No	704 (79.9)	164 (73.9)	169 (76.8)	179 (81.4)	192 (87.7)	
Yes	177 (20.1)	58 (26.1)	51 (23.2)	41 (18.6)	27 (12.3)	
Fatty liver, n (%)						< 0.001
No	628 (71.3)	217 (97.7)	196 (89.1)	151 (68.6)	64 (29.2)	
Yes	253 (28.7)	5 (2.3)	24 (10.9)	69 (31.4)	155 (70.8)	
METS-IR	33.5 ± 6.5	26.0 ± 1.9	30.7 ± 1.2	35.1 ± 1.4	42.3 ± 4.4	< 0.001

Data were mean ± SD or median (IQR) for skewed variables or numbers (proportions) for categorical variables.

BMI, body mass index; SBP, systolic blood pressure; DBP, diastolic blood pressure; ASL, aspartate aminotransferase; ALT, alanine aminotransferase; GGT, γ-glutamyltranspeptidase; FPG, fasting plasma glucose; UA, uric.acid; TC, total cholesterol; TG, triglyceride; HDL-c, high‐density lipoprotein cholesterol; LDL-C, low-density lipoprotein cholesterol; METS-IR, metabolic score for insulin resistance; Q1, Q2, Q3, and Q4 are quartiles of the metabolic score for insulin resistance(METS-IR).

### 3.2 Univariate and multivariate analyses of METS-IR and eGFR

As shown in **Table 2**, the univariate analysis indicated that METS-IR per 10-unit rise, sex, age, BMI, SBP, DBP, GGT, FPG, uric acid, TC, TG, HDL-c, LDL-c, fatty liver, and smoking status were associated with eGFR (all P-values < 0.05). The eGFR level decreased by 2.8 mL/min (95% CI: -4 to -1.59) for every 10-unit rise in METS-IR.

**Table 2:** Results of univariate analysis of eGFR.

Variable	β (95%CI)	*p* value
METS-IR per 10	-2.8 (-4,-1.59)	< 0.001
Sex, n (%)	3.11 (1.46,4.77)	< 0.001
Age, (years)	-0.51 (-0.59,-0.43)	< 0.001
BMI, (kg/m2)	-0.37 (-0.62,-0.12)	0.004
SBP, (mmHg)	-0.12 (-0.17,-0.07)	< 0.001
DBP, (mmHg)	-0.21 (-0.29,-0.14)	< 0.001
AST, (IU/L)	-0.09 (-0.18,0.01)	0.087
ALT, (IU/L)	-0.03 (-0.08,0.03)	0.311
GGT, (IU/L)	-0.04 (-0.07,-0.01)	0.021
FPG, (mg/dl)	-0.15 (-0.2,-0.09)	< 0.001
Uric acid, (mg/dl)	-2.67 (-3.22,-2.12)	< 0.001
TC, (mg/dl)	-0.06 (-0.08,-0.03)	< 0.001
TG, (mg/dl)	-0.03 (-0.04,-0.02)	< 0.001
HDL-c, (mg/dl)	0.09 (0.03,0.14)	0.002
LDL, (mg/dl)	-0.06 (-0.09,-0.04)	< 0.001
Habit.of.exercise, n (%)	-1.25 (-3.23,0.72)	0.214
Fatty liver, n (%)	-1.97 (-3.72,-0.22)	0.027
Alcohol consumption	-1.68 (-3.7,0.34)	0.103
Smoking status	2.31 (0.39,4.23)	0.018

METS-IR, metabolic score for insulin resistance; eGFR, estimated glomerular filtration rate; BMI, body mass index; SBP, systolic blood pressure; DBP, diastolic blood pressure; AST, aspartate aminotransferase; ALT, alanine aminotransferase; GGT, γ-glutamyltranspeptidase; FPG, fasting plasma glucose; UA, uric.acid; TC, total cholesterol; TG, triglyceride; HDL-c, high‐density lipoprotein cholesterol; LDL-C, low-density lipoprotein cholesterol.

**Figure 2:**
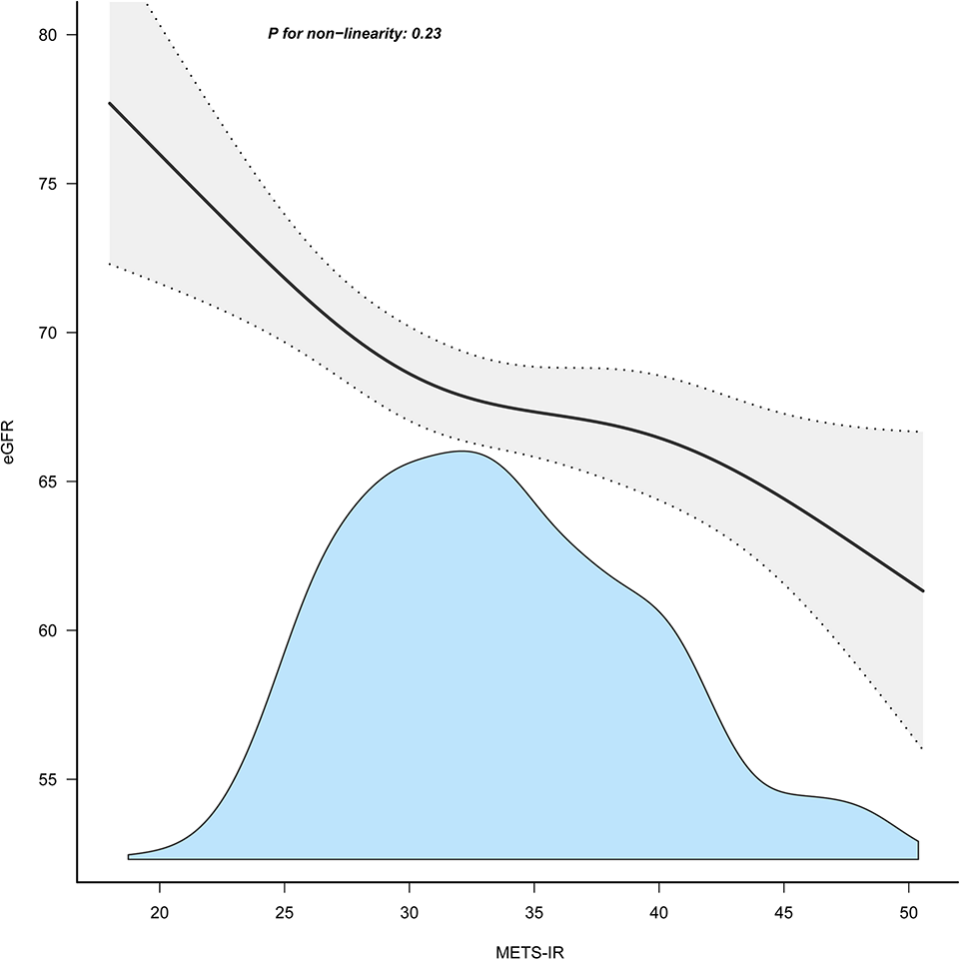
Association between METS-IR index and eGFR. METS-IR, metabolic score for insulin resistance; eGFR, estimated glomerular filtration rate.

**Table 3** displays the results of multivariable regression analyses. All four models showed a negative correlation between METS-IR and eGFR level after controlling for different potential confounders (**Table 3**). A decrease in eGFR of 2.54 units was reported for every 10-unit rise in METS-IR (adjusted β = -2.54, 95% CI: -4.04 to -1.05, P = 0.001) when METS-IR was used as a continuous variable in the fully adjusted model (model 3). When METS-IR was converted into categorical variables according to quartiles, compared with that of participants with lower METS-IR Q1 (≤ 28.61), the adjusted β values for METS-IR and eGFR in Q2, Q3, and Q4 were -3.32 (95% CI: -5.36 to -1.28, P = 0.001), -3.51 (95% CI: -5.73 to -1.28, P = 0.002), and -5.04 (95% CI: -7.65 to -2.43, P < 0.001), respectively, in the fully adjusted model. In addition, as METS-IR increased, the eGFR levels of the study participants showed a decreasing trend (**Table 3**, P for trend < 0.001). Furthermore, we found an increasing trend of a significant negative linear correlation between the METS-IR index and eGFR (**Figure 2**).

**Table 3:** Multivariable-adjust β and 95%CI of the METS-IR index quartiles associated with eGFR.

Variable	unadjusted		model1		model2		model3	
	β (95%CI)	*P*_value	β (95%CI)	*P*_value	β (95%CI)	*P*_value	β (95%CI)	*P*_value
METS-IR per 10	-2.8 (-4~-1.6)	<0.001	-2.63 (-3.81~-1.46)	<0.001	-2.21 (-3.59~-0.84)	0.002	-2.54(-4.04~-1.05)	0.001
1st Quartile(≤28.61)	Ref		Ref		Ref		Ref	
2st Quartile(28.61-32.84)	-4.95 (-7.14~-2.76)	<0.001	-3.61 (-5.63~-1.59)	<0.001	-3.34 (-5.38~-1.31)	0.001	-3.32 (-5.36~-1.28)	0.001
3st Quartile(32.84-37.62)	-5.18 (-7.37~-2.99)	<0.001	-3.9 (-6.01~-1.78)	<0.001	-3.36 (-5.55~-1.17)	0.003	-3.51 (-5.73~-1.28)	0.002
4st Quartile(≥37.62)	-6.27 (-8.46~-4.07)	<0.001	-5.51 (-7.66~-3.37)	<0.001	-4.63 (-7.04~-2.21)	<0.001	-5.04 (-7.65~-2.43)	<0.001
p for trend		<0.001		<0.001		<0.001		<0.001

Model 1 adjust for age and sex.

Model 2 adjust for Model 1+ SBP, DBP, ALT, FPG.

Model 3 adjust for Model 1+ Model 2 +parameters of lifestyles (Exercise, Fatty liver, Alcohol consumption, Smoking status)

METS-IR, metabolic score for insulin resistance; eGFR, estimated glomerular filtration rate; Ref, reference; SBP, systolic blood pressure; DBP, diastolic blood pressure; ALT, alanine aminotransferase; FPG, fasting plasma glucose.

### 3.3 Subgroup analysis

**Figure 3:**
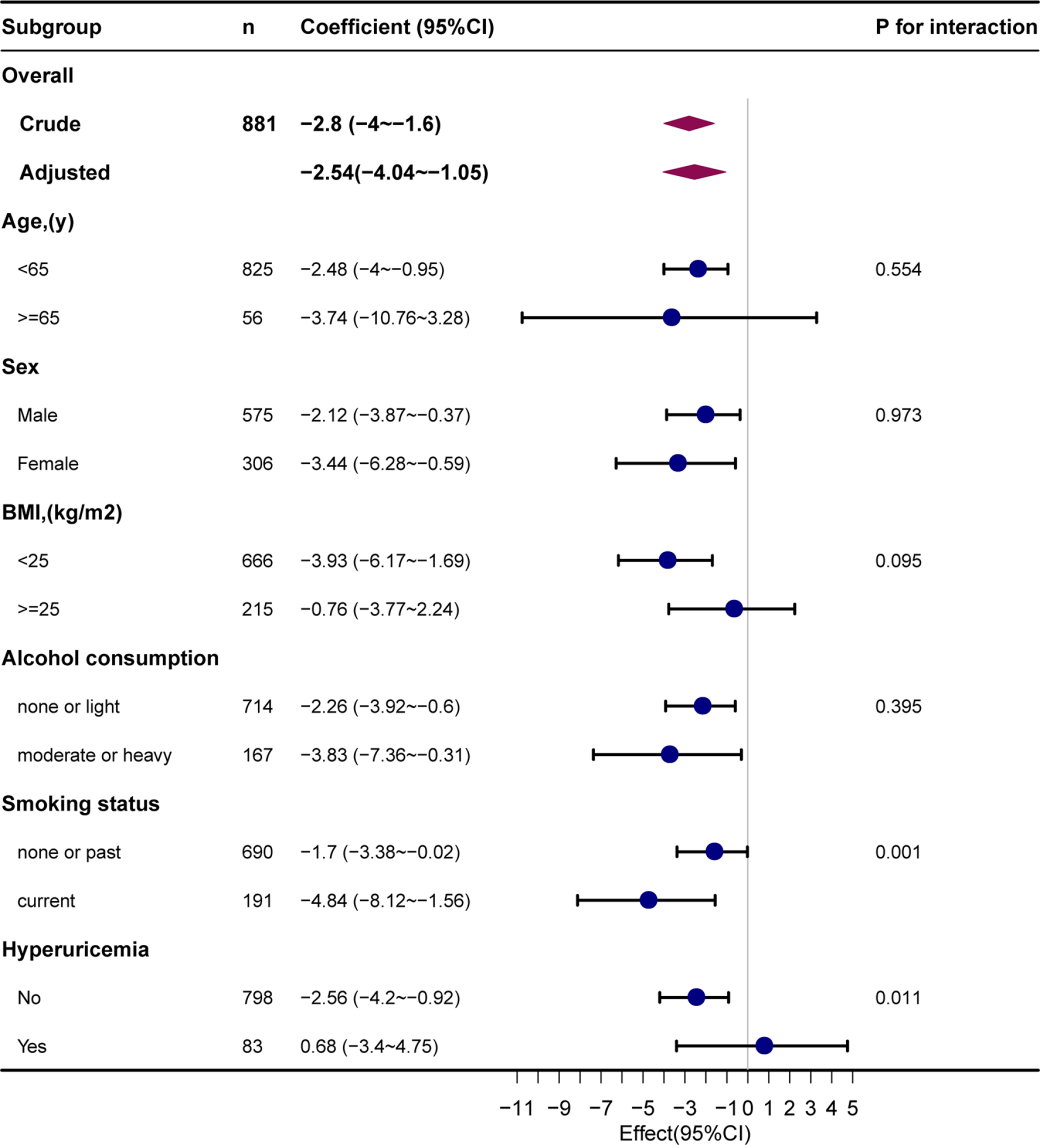
Subgroup analyses of the METS-IR and eGFR. METS-IR, metabolic score for insulin resistance; eGFR, estimated glomerular filtration rate.

The results of the subgroup analysis are presented in **Figure 3**. Stratified analysis suggested no marked interaction between METS-IR and eGFR across age (< 65 or ≥ 65 years), sex (male or female), BMI (< 25 or ≥ 25 kg/m2), and alcohol consumption (none or light, moderate or heavy) groups. The association between METS-IR and eGFR was robust across age (P for interaction = 0.554), sex (P for interaction = 0.973), BMI (P for interaction = 0.095), and alcohol consumption (P for interaction = 0.395) subgroups. In addition, we observed a significant interaction in the subgroups of smoking status (P for interaction = 0.001) and HUA (P for interaction = 0.011). The correlation between METS-IR and eGFR was statistically stronger in smokers (β = -4.84, 95% CI, -8.12 to -1.56) and non-hyperuricemic individuals (β = -2.56; 95% CI, -4.2 to -0.92). In addition, we observed a significant interaction in the subgroups of smoking status (P for interaction = 0.001) and HUA (P for interaction = 0.011). The correlation between METS-IR and eGFR was statistically stronger in smokers (β = -4.84, 95% CI, -8.12 to -1.56) and non-hyperuricemic individuals (β = -2.56; 95% CI, -4.2 to -0.92).

## 4 Discussion

In our cross-sectional analysis, the METS-IR index, as a categorical/continuous variable, was negatively correlated with eGFR after adjusting for other covariates among individuals who underwent health check-ups in Gifu, Japan. The results of the subgroup analysis were stable across age, sex, BMI, and alcohol consumption groups. Interestingly, the association of METS-IR with eGFR was significantly different in the smoking status and HUA subgroups.

METS-IR is a recently developed score to evaluate IR that is not dependent on insulin measures but rather on laboratory tests (such as lipids and blood glucose) that are readily available in primary healthcare (13, 17). HEC (13, 14), the best way to measure IR, is invasive and not good for a broad population. Among the most popular IR indicators used in clinical epidemiology research is the “HOMA-IR,” derived from an insulin measurement (15, 17). However, because of its price, especially in less developed areas, its practical applicability is constrained (17, 34). METS-IR is a novel alternative to IR. It is calculated using standard measurements (FPG, TG, HDL, and BMI), and studies have shown that it agrees well with the results of HEC and intravenous glucose tolerance (13, 17). Therefore, METS-IR has been promoted in recent years for the assessment of IR.

The relationship between IR and CKD had been demonstrated in previous studies (7–9, 12). Irrespective of diabetes prevalence, a rising trend in HOMA-IR was related to an increased risk of unfavorable renal outcomes during the course of a 12-year prospective cohort study of the general population without CKD (hazard ratio = 2.06, 95% CI: 1.62–2.60, *P* < 0.001) (7).

However, some studies failed to find a significant relationship between eGFR and IR. Johns et al. (35) observed no connection between IR and eGFR, even though patients with MS had a greater risk of CKD and a lower average eGFR than that of the others. Only hypertension was found to be correlated to CKD (odds ratio = 3.5, 95% CI: 1.2–10.1, *P* = 0.02). In another former study (36), multivariate analysis found no significant connection between HOMA-IR index values and eGFR. Analysis of covariance-adjusted HOMA-IR index values in lower eGFR groups were not substantially higher. They found no notable variations in HOMA-IR values by the number of MS components. They concluded that reduced renal function is unrelated to IR. There are also other studies that have made similar conclusions (37–39). The possible explanation is that our study differs from theirs in the study population and the metrics used to proxy for IR. The study population of this study was a healthy physical examination population, without deliberate exclusion of the CKD population, which is closer to the real world. Furthermore, METS-IR, as a new IR alternative marker, can respond to IR even better than that of HOMA-IR (13, 40).

The results of our study are in agreement with those found by Shi W et al. (41). TyG is another novel alternative marker of IR. Shi W et al. reported that after full adjustment, each SD increase in TyG resulted in an extra 42.6 percent probability of lower eGFR. The highest TyG quartile had 1.934 times the risk of the lowest. The eGFR decreased linearly with TyG. This study indicated TyG as a predictive tool for preventing low eGFR (41). Takuro Okamura et al. further reported that the TyG index presented a significant risk of developing CKD in all study participants and demonstrated that the TyG index could be used as a predictor for incident CKD (16). According to recent research by Pengbo Wang et al., a high METS-IR score was linked to an increased risk of slightly lower eGFR and a high risk of quick eGFR decline in the Chinese rural population (excluding those with eGFR less than 60 mL/min) (8). Our findings are consistent with the above studies.

Both our study and prior research have investigated the connection between METS-IR and eGFR, and the pathophysiological basis for this result is strong. It has been previously shown that eGFR and other elements of the MS help to explain the variability of IS in older men with CKD stages 3 and 4 (42). Various probable mechanisms connecting the IR and the development of CKD have been revealed by some investigations, meantime. IR has various effects on kidney function, including abnormalities in hemodynamics, podocyte function, and tubular function, among others (43). They hypothesized that endothelial cell injury and enhanced vascular permeability induced by IR or hyperinsulinemia would lead to glomerular ultrafiltration and mesenteric hyperproliferation, which would then lead to a decrease in eGFR (44–46). On the other hand, other investigations found that overactivated inflammatory responses could govern elevated IR states. Therefore, the pathophysiology of CKD is heavily influenced by oxidative stress, the inflammation process, and metabolic acidosis brought on by IR (16).

Additionally, we noticed a significant interaction in the relationship between the METS-IR index and eGFR in the subgroups of smoking status and HUA, respectively. We observed that this association was more pronounced in the smoking population. According to a previous community study, which supports this idea, those who smoke cigarettes regularly have relatively low IS than that of those non-smokers, and IS increases after 1–2 weeks of quitting smoking, although it does not return to normal (47). We also found that METS-IR was meaningfully correlated with eGFR in individuals without HUA, whereas in individuals with HUA, this association was absent. The possible reason is that METS-IR is strongly associated with HUA based on a previous study (32, 33), which weakened its association with eGFR. The above findings require further research.

This study, however, had some limitations. First, our study was cross-sectional, which makes causal inferences from our findings more difficult. Second, we did not assay HOMA-IR, which has been more widely used in past research to assess IR. However, in previous studies comparing the validation of multiple metrics, HOMA-IR and METS-IR were included to assess IR levels, and their similarity in different clinical settings was noted (13, 40). The conclusions showed that METS-IR and HOMA-IR had similar assessment effects. Third, the detailed methods used in the medical examination, such as blood pressure measurements as well as original data of serum creatinine, height, and weight, are not clear because the data used in this study came from other studies that were already published. Fourth, just like in any observational study, it is possible that unmeasured confounders played a role in the detected association between METS-IR and eGFR. Finally, because almost all of the participants were Japanese, it is not clear whether our conclusions are applicable to other ethnic groups.

## 5 Conclusions

The METS-IR is highly associated with eGFR among individuals who underwent health check-ups in Gifu, Japan. These findings indicated that individuals with elevated METS-IR should have closer monitoring of their renal function and be identified in time to avoid the eventual development of renal failure. METS-IR might be employed as a monitoring marker for early screening, primary prevention, and diagnostic and therapy management techniques for CKD; however, further research is needed to prove it.

## Data availability statement

The original contributions presented in the study are included in the article/supplementary material. Further inquiries can be directed to the corresponding author.

## Ethics statement

The studies involving human participants were reviewed and approved by the ethics committee of Murakami Memorial Hospital. The patients/participants provided their written informed consent to participate in this study.

## Author contributions

The research was designed by GLL and FS. GLL, LT, QZ, and LY gathered and analyzed the data. GLL wrote the first version of the manuscript. GLL, XJ, and FS helped improve the manuscript. All authors contributed to the article and approved the submitted version.
